# Development of human brain neuroimmune system under influence of alcohol

**DOI:** 10.1192/j.eurpsy.2022.1640

**Published:** 2022-09-01

**Authors:** T. Shushpanova, A. Solonskii, S. Shumilova, O. Shushpanova, N. Bokhan

**Affiliations:** 1 Mental Health Research Institute of Tomsk National Investigation MedicalCenter of Russian Academy of Sciences, Clinical Psychoneuroimmunology And Neurobiology, Tomsk, Russian Federation; 2 Siberian State Medical University, Histology, Embryology And Cytology, Tomsk, Russian Federation; 3 Mental Health Research Centre, Department Of Childhood Psychiatry, Moscow, Russian Federation; 4 Siberian State Medical University, Department Of Psychiatry, Narcology And Psychotherapy,, Tomsk, Russian Federation; 5 Mental Health Research Institute Tomsk National Research Medical Center Russia Academy of Science, , Department Of Addictive States, Tomsk, Russian Federation; 6 Siberian State Medical University, Psychiatry, Narcology, Psychotherapy, Tomsk, Russian Federation

**Keywords:** embryogenesis, neuroimmune system, brain, alcohol, glioblast, GABAA receptor, synapse

## Abstract

**Introduction:**

Exposure to alcohol causes imbalances in neuroimmune function and impaired brain development.

**Objectives:**

Alcohol activates neuroimmune molecules, expressed and secreted by glial cells in the brain, alter neuronal function and stimulate alcoholic behavior.

**Methods:**

The study involved women aged 25-41 years-did not drink alcohol 1 month before and during pregnancy – 1-st group; women with I-II degree of alcoholism 3-13 years – 2-nd group. Embryonic material were obtained 8-15 weeks of igestation. 2-nd group were divided into subgroups. Group Alcohol (A)-alcoholic women,s embrious, included 2 subgroups: A1-embryos 8-9 weeks, A2-10-11 weeks of gestation (n=12). The Control group (K) includ control samples K1-8–9, K2-10-11 weeks (n=14). The analysis of changes in morphometric parameters was used to identify quantitative changes among glioblasts, correlation between the degree of differentiation components and the degree of influence of alcohol. For this, the program AxioVision 4.8 was used Parameters of GABAA/benzodiazepine receptors were studied by the radio-receptor assay of [3H]-flunitrazepam with synaptoneurosomes.

**Results:**

Changes in glioblasts tof human brain embryos and fetuses were revealed under conditions of chronic prenatal alcoholization with an increase in gestational age compared to the control subgroups: a significant increase in the average number of glioblasts, the length of the perimeters of the presynaptic terminal, postsynaptic density, presynaptic terminal areas were significantly less (p<0, 01) in the study group than in the control. Exposure to ethanol reduces the affinity of GABAA/benzodiazepine receptors, which affects neuronal plasticity associated with the development of glioblasts and neuroblasts during embryogenesis.

**Conclusions:**

Changes in microglial cause disruption of the neuronal activity

**Disclosure:**

No significant relationships.

